# Predictive Value of Uric Acid Regarding Cardiometabolic Disease in a Community-Dwelling Older Population in Shanghai: A Cohort Study

**DOI:** 10.3389/fmed.2020.00024

**Published:** 2020-02-05

**Authors:** Qin Lan, Hong Wu, Xiaohui Zhou, Liang Zheng, Fang Lin, Qingshu Meng, Xiaoling Xi, Aixue Yue, Nicholas Buys, Jing Sun, Zhongmin Liu, Jue Li, Huimin Fan

**Affiliations:** ^1^Shanghai East Hospital, Tongji University, Shanghai, China; ^2^School of Medicine, Tongji University, Shanghai, China; ^3^Gaohang Community Hospital, Pudong New Area, Shanghai, China; ^4^Menzies Health Institute Queensland, Griffith University, Gold Coast, QLD, Australia; ^5^School of Medicine, Griffith University, Gold Coast, QLD, Australia

**Keywords:** uric acid, cardiometabolic disease, community older population, predictor, cohort study

## Abstract

**Aim:** This study aimed to test the predictive power of serum uric acid (UA) levels on new-onset cardiometabolic risk in the Chinese population.

**Methods:** Older people who visited a community health center for a yearly health check (*N* = 5,000; men: 47%, women: 53%) were enrolled. Participants were followed for 4 years from baseline (median: 48 months), with the endpoints being development of heart failure, atrial fibrillation, diabetes, hypertension, metabolic syndrome, or kidney disease.

**Results:** During follow-up, 342 men (7.4%) and 360 women (8.6%) developed hypertension; 98 men (2.48%) and 135 women (3.06%) developed diabetes; and 175 men (5.04%) and 214 women (4.51%) developed metabolic syndrome. Incident diabetes, hypertension, and metabolic syndrome increased with increased UA levels at baseline (*P* < 0.001). A multivariate Cox proportional hazards analysis revealed a significant, independent association between the baseline UA level and the onset and future hypertension and/or diabetes in both men and women. However, UA is associated with the development of metabolic syndrome in men, but not in women.

**Conclusion:** UA is an independent predictor of new-onset diabetes and hypertension in both women and men and a predictor of new-onset metabolic syndrome only in men.

## Introduction

Lifestyle changes have been shown to be a significant factor in the increasing prevalence of hyperuricemia, and several studies have indicated that uric acid (UA) is a risk factor for cardiometabolic disease (e.g., hypertension, diabetes mellitus, chronic kidney disease, coronary heart disease or stroke) in clinical settings (1–5). A possible reason for this increase is the gradual change in dietary intake toward high fat, protein, and carbohydrate consumption, which leads to increasing UA levels (6).

It is well-established that older people are at increased risk of cardiometabolic disease (7, 8). In particular, the reduction in renal function that occurs with aging is associated with an increased number of cardiometabolic disease events independent of other confounding factors, such as age and metabolic conditions (9). Moreover, the incidence of cardiometabolic disease has also been shown to increase with increasing UA levels (10).

Previous studies have shown that high UA levels are associated with hypertension. However, this studies have not comprehensively assessed the association between hyperuricemia and cardiometabolic disease, including stroke, coronary disease, and cardiovascular events. In addition, most of these studies were conducted at least 10 years ago. Therefore, owing to the possibility of shared genetic factors for hyperuricemia and cardiometabolic disease, whether hyperuricemia is an independent risk factor for adult cardiometabolic disease remains unclear. Moreover, the relationship between serum UA levels and cardiometabolic disease has not been studied in the Chinese population in China.

Gaohang is a large district in Pudong New Territory in Shanghai Municipal City, which is located in the central part of the east coast of China. Shanghai East Hospital is responsible for the healthcare of the Gaohang community, and the hospital's Heart Disease Research Group has built health records from July 2013 to December 2017 for all people of this community. Hence, a prospective study was conducted to examine the effect of serum UA on the incidence of cardiometabolic disease in this cohort.

## Methods

### Sample

The Gaohang community cohort includes 5,000 older people who were randomly selected from 137,625 residents, who are mainly Han Chinese. The participants underwent a health screen at Shanghai East Hospital between 1 March 2013 and 30 June 2013 and follow-up visits in June 2014 and December 2017. At the baseline and at the 2014 and 2017 follow-up visits, 3,204 people, accounting for 64.08% retention rate, completed the medical screening and biological tests and answered the survey questions. A comparison between the 3,204 participants and the 1,796 participants who dropped out of the study did not show differences in age, gender, education, marital status, or occupation. Participants with an established cardiometabolic disease or without available data on UA were excluded. The Ethics Committee of Shanghai East Hospital approved the study, and all participants provided consent for their participation in the study.

### Demographic Characteristics

Data on participants' demographic characteristics and on their health behavior, diet, disease history, medication use, and treatment for cardiometabolic disease were collected using a survey. Their weight and body mass index (BMI) were measured during the health examination. BMI was defined as weight in kilograms divided by the square of the height in meters (kg/m^2^). Disease history was defined as a history of cardiometabolic disease, which includes one or all of the following: hypertension, diabetes mellitus, chronic kidney disease, coronary heart disease, and stroke. Medication use was defined as any medication prescribed by doctors for the treatment of cardiometabolic disease.

Exercise data were collected using a questionnaire and were classified as no exercise, mild exercise, and moderate exercise according to the Physical Exercise Guideline (11). No exercise includes sitting, lying, and no oxygen consumption-related activities. Examples of mild exercise include walking slowly (e.g., shopping and walking around office), preparing food, and washing dishes. Examples of moderate exercise include walking briskly, slow dancing, vacuuming, and playing badminton and basketball. Smoking was defined as daily consumption of at least one cigarette for at least 1 year, and drinking was defined as >50 g average daily consumption of alcohol for more than 1 year.

### Laboratory Measurements

Prior to their laboratory tests, the participants were instructed to fast for at least 12 h. Their BP was measured using a calibrated mercury sphygmomanometer after they had rested for at least 5 min. The BP was measured three times continuously, with a break of 1–2 min between measurements, and the average of the three measurements was considered the final BP.

Samples of the concentrations of serum UA, fasting glucose (FBG), total cholesterol (TC), triglycerides (TG), high-density lipoprotein (HDL), low-density lipoprotein (LDL), serum creatinine (Cr), and C-reactive protein (CRP) were coded and stored at −80 CO until analysis. Laboratory technicians conducted a blinded analysis of the samples. Serum UA, glucose, TC, TG, LDL, Cr, and CRP concentrations were measured using standard enzymatic automated methods. HDL-c was measured through the enzymatic procedure after precipitation. All examinations were repeated during follow-up visits in 2014 and 2017.

### Definition of Disease and Diagnostic Criteria

Heart failure, heart disease events, and atrial fibrillation are defined by the American Heart Association guidelines (1). Hypertension is defined as having systolic BP ≥ 140 mmHg and/or diastolic BP ≥ 90 mmHg according to the 2017 American Heart Association Guidelines for people aged below 65 years, and SBP ≥ 150 mmHg and/or DBP ≥ 100 mmHg for people aged 65 years and above (1). Diagnosis of metabolic syndrome was defined according to 2017 American Heart Association Guidelines (12). If an individual met three of the five criteria, the condition was diagnosed as metabolic syndrome: Asian: waist circumference more than 100 cm in men or more than 85 cm in women (population-specific modifications); TG > 150 mg/dL, HDL-C <40 mg/dL in men or <50 mg/dL in women or on drug treatment; high BP: > 130 mm Hg systolic or > 85 mm Hg diastolic or on antihypertensive treatment; high glycemia: more than 100 mg/dL in glucose or on drug treatment for elevated glucose. Stroke, which was defined according to the World Health Organization definition (that was introduced in 1970 and is still used), is “rapidly developing clinical signs of focal (or global) disturbance of cerebral function, lasting more than 24 h or leading to death, with no apparent cause other than that of vascular origin” (13). Kidney disease was defined as kidney damage or a GFR below 60 mL per minute per 1.73 m^2^ for 3 months or more (14). Diabetes was defined based on the parameters established by the China Diabetes Society, namely, FBG of 7.0 mmol/L or more (15). Diagnoses of heart failure, heart disease events, atrial fibrillation, diabetes, hypertension, metabolic syndrome, and kidney disease were confirmed by cardiac specialists based on the pathological diagnosis, ECG, and ultrasound tests.

### Statistical Analysis

Data analysis was performed using SPSS 25.0 (SPSS, Chicago, IL, USA). Numeric data were expressed as mean ± standard deviation (SD), whereas categorical data were expressed as numbers and percentages. To identify potential confounding factors, the chi-square test (χ^2^) was used to compare the differences between the group with disease and the group without disease in categorical variables, including age, occupation, education, marital status, income, BMI, smoking, drinking, and exercise. An independent *t*-test was used to compare the differences between these two groups in the continuous variables, including waist circumference, systolic BP, diastolic BP, TC, LDL, HDL, glucose, UC, UN, and CRP in the 2013 baseline data. If any statistical differences in any of these factors were found, these were subsequently included in the multivariate analysis using the Cox regression analysis. The *t*-tests were used to test the differences between the groups with and without disease. The chi-square test and *t*-tests were conducted on male as well as female participant groups.

The baseline serum UA was further divided into four quartiles, and the association between UA and each laboratory measure was analyzed using analysis of variance (ANOVA) to examine whether the laboratory-based measures had increased with increasing UA levels in men and women. The prediction of serum UA on each of the diseases was conducted using the Cox regression analysis on data for the male and female participant groups. The normality of all continuous variables was checked prior to the analyses to ensure they met the statistical assumptions. *P* < 0.05 were considered statistically significant for the bivariate analysis and <0.01 for the Cox regression analysis when confounding factors were included in the analyses. The time-to-event was calculated as person-years data when participants did not have any diseases and had incidence of the disease condition in the follow-up years, that is, in either 2014 or 2017.

## Results

The baseline characteristics of the participants are shown in Table 1.

**Table 1:** Comparison between disease group and normal group in 2013 (disease are defined as cases from 2014 and 2017 results).

	**Total**			**Male**			**Female**		
**Variables**	**Disease group**	**Normal group**	**Statistics**	**Disease group**	**Normal group**	**Statistics**	**Disease group**	**Normal group**	**Statistics**
	**(*n* = 371)**	**(*n* = 2,833)**	***p***	**(*n* = 142)**	**(*n* = 1,237)**	***p***	**(*n* = 229)**	**(*n* = 1,596)**	***p***
**Gender:** ***n*** **(%)**									
Female	233 (62.8)	1590 (56.1)	**0.02**						
Male	138 (37.2)	1243 (43.9)							
**Age (years):** ***n*** **(%)**									
≤ 65	72 (19.4)	533 (18.8)	0.80	25 (17.6)	244 (19.7)	0.71	47 (20.5)	289 (18.1)	0.20
66–70	126 (34.0)	979 (88.6)		48 (33.8)	439 (35.5)		**78 (34.1)**	**540 (33.8)**	
71–75	63 (17.0)	545 (19.2)		32 (22.5)	237 (19.2)		31 (13.5)	308 (19.3)	
76–80	68 (18.3)	471 (16.6)		20(14.1)	198 (16.0)		48 (21.0)	273 (17.1)	
>80	42 (11.3)	305 (10.8)		17 (12.0)	119 (9.6)		25 (10.9)	186 (11.7)	
**Occupation:** ***n*** **(%)**									
Office worker	53 (14.3)	438 (89.2)	0.11	29 (20.4)	280 (22.7)	0.20	24 (10.5)	158 (9.9)	0.78
Operator	115 (31.1)	1021 (89.9)		67 (47.2)	636 (51.5)		48 (21.1)	385 (24.2)	
Farmer	181 (48.9)	1195 (86.8)		36 (25.4)	221 (17.9)		145 (63.6)	974 (61.1)	
Others	21 (5.7)	174 (6.2)		10 (7.0)	97 (7.9)		11 (4.8)	77 (4.8)	
**Education:** ***n*** **(%)**									
Primary below	87 (23.6)	537 (19.0)	0.09	15 (10.6)	60 (4.9)	**0.03**	72 (31.7)	477 (29.9)	0.13
Primary school	75 (20.3)	621 (21.9)		22 (15.5)	239 (19.3)		**53 (23.3)**	**382 (24.0)**	
Secondary school	165 (44.7)	1402 (49.5)		80 (56.3)	735 (59.5)		85 (37.4)	667 (41.8)	
College and above	42 (11.4)	270 (9.5)		25 (17.6)	202 (16.3)		17 (7.5)	68 (4.3)	
**Marital:** ***n*** **(%)**									
No married	83 (22.5)	638 (22.6)	0.96	17 (12.0)	132 (10.7)	0.65	66 (29.1)	506 (31.8)	0.41
Married	286 (77.5)	2183 (77.4)		125 (88.0)	1098 (89.3)		161 (70.9)	1085 (68.2)	
**Monthly income (**¥**):** ***n*** **(%)**									
<1,500	97 (26.6)	759 (27.2)	0.99	21 (15.1)	185 (15.2)	0.70	76 (33.8)	574 (36.5)	0.82
1,500–2,000	64 (1.6)	503 (18.0)		22 (15.8)	237 (19.4)		42 (18.7)	266 (16.9)	
2,000–2,500	87 (23.9)	654 (23.4)		33 (23.7)	297 (24.3)		54 (24.0)	357 (22.7)	
> 2,500	116 (31.9)	878 (31.4)		63 (45.3)	501 (41.1)		53 (23.6)	377 (24.0)	
**Medication taking:** ***n*** **(%)**									
No	306 (88.7)	886 (33.1)	**<0.001**	114 (87.7)	420 (35.9)	**<** **0.001**	192 (89.3)	466 (31.0)	**<** **0.001**
1 type medication and more	39 (11.3)	1788 (66.9)		16 (12.3)	751 (64.1)		23 (10.7)	1037 (69.0)	
**Smokin** **g:** ***n*** **(%)**									
No	306 (82.5)	2164 (76.4)	**0.002**	80 (56.3)	600 (48.5)	**0.009**	226 (98.7)	1564 (98.0)	
Yes	49 (13.2)	393 (13.9)		47 (33.1)	372 (30.1)		2 (0.9)	21 (1.3)	
Smoked before but stopped	16 (4.3)	276 (9.7)		15 (10.6)	265 (21.4)		1 (0.4)	11 (0.7)	
**Exercise intensity:** ***n*** **(%)**									
No exercise	17 (4.7)	134 (4.9)	0.06	6 (4.3)	44 (3.7)	0.43	11 (5.0)	90 (5.9)	0.06
Light-intensity	229 (63.6)	1876 (69.2)		84 (60.9)	793 (66.4)		145 (65.3)	1083 (71.4)	
Moderate-intensity	114 (31.7)	700 (25.8)		48 (34.8)	357 (29.9)		66 (29.7)	343 (22.6)	

*WC, Waist circumference; ARB, Angiotensin receptor blocker; DBP, Diastolic blood pressure; SBP, Systolic blood pressure; IVS, Interventricular septum; ALT, Alanine aminotransferase; LAD, Left atrial diameter; LVDS, left ventricular end-systolic diameter; LVDD, Left ventricular end-diastolic diameter*.

*Disease group includes heart disease, cases with heart disease events, atrial fibrillation, diabetes, hypertension, metabolic syndrome and kidney disease. These diseases are also listed in Table 3. Bold values are statistically significant*.

The overall mean baseline UA was 5.96 (1.39) mg/dl in men and 5.07 mg/dl in women in 2013 and 6.05 (1.55) mg/dl in men and 5.21 (1.34) mg/dl in women in 2017. Participants were divided into eight groups based on the quartiles of the baseline serum UA levels. The mean baseline serum UA in the male quartile groups was 4.25, 5.25, 5.80, and 6.57 mg/dl, and in the female quartile groups was 3.80, 4.89, 5.79, and 7.36 mg/dl. In 2017, the mean serum UA in the male quartile groups was 4.32, 5.36, 6.04, and 7.12 mg/dl, and in the female quartile groups was 4.25, 5.25, 5.80, and 6.57 mg/dl.

Table 2 shows the association of SBP, TG, TC, HDL, LDL, Glucose Cr, CRP, and BMI, with UA and this was further analyzed using ANOVA. As shown in Table 2, systolic BP, BMI, TG, TC, HDL, LDL, Cr, and BMI all increased with increasing levels of serum UA in both men and women. These characteristics were significantly different among the quartiles (*P* < 0.001); these factors were considered confounding factors and were included in the next step, the Cox regression analysis. In the ANOVA analysis, age and diastolic BP were not significantly associated with the increasing levels of serum UA in men, and TC and glucose were not significantly associated with the increasing levels of UA in women. Glucose was not associated with the increasing levels of UA in either men or women. Hence, age and diastolic BP were not included in the Cox regression analysis for men and TC and glucose were not included in that for women.

**Table 2:** Comparison between characteristics by categories of serum uric acid of baseline (uric acid is from baseline).

**Variables**	**Serum uric acid quartile(mg/dL) (Men)** ***n*** **=** **1,379**					**Serum uric acid quartile(mg/dL) (Women)** ***n*** **=** **1,825**				
	**≤4.52**	**4.53–5.34**	**5.35–6.33**	**≥6.34**	**F(p)**	**≤4.52**	**4.53–5.34**	**5.35–6.33**	**≥6.34**	**F(p)**
Uric Acid in 2013	4.25 (0.93)	5.25 (0.94)	5.80 (1.14)	6.57 (1.39)	1051.04 (**0.001**)	3.80 (0.54)	4.89 (0.23)	5.79 (0.28)	7.36 (0.96)	1837.71 (**<0.001**)
Uric Acid in 2017	4.31.13)	5.36 (0.83)	6.04 (1.17)	7.12 (1.48)	142.89 (**0.001**)	4.25 (0.93)	5.25 (0.94)	5.80 (1.14)	6.57 (1.40)	200.32 (**0.001**)
Age: M (SD)	71.61 (6.32)	71.38 (6.30)	71.54 (6.29)	72.15 (6.67)	1.46 (0.22)	71.04 (6.25)	71.80 (6.63)	72.61 (6.71)	74.27 (7.04)	20.98 (< **0.001**)
Body mass index: M (SD)	23.61 (3.18)	23.80 (3.23)	24.57 (2.96)	25.14 (2.99)	22.46 (< **0.001**)	23.56 (3.34)	24.92 (3.34)	25.34 (3.66)	26.05 (3.74)	51.62 (< **0.001**)
Waist circumferences (cm): M (SD)	85.42 (9.16)	85.73 (9.74)	88.02 (8.34)	90.18 (8.13)	28.67 (< **0.001**)	82.77 (8.49)	85.69 (8.90)	87.26 (9.21)	89.47 (9.35)	53.87 (< **0.001**)
Systolic blood pressure (mmHg): M (SD)	136.31 (16.55)	136.96 (17.64)	138.31 (17.17)	139.73 (17.46)	3.22 (**0.02**)	137.16 (16.94)	139.59 (17.73)	139.96 (17.08)	142.19 (17.05)	7.60 (< **0.001**)
Diastolic blood pressure (mmHg): M (SD)	81.21 (8.69)	82.49 (8.51)	82.48 (9.32)	82.99 (9.45)	2.10 (0.10)	80.47 (8.60)	81.60 (8.68)	82.02 (8.58)	81.88 (8.85)	4.27 (**0.005**)
Total cholesterol (mmol/L): M (SD)	4.73 (0.99)	4.59 (0.84)	4.78 (0.89)	4.79 (0.95)	4.44 (**0.004**)	5.17 (0.97)	5.15 (0.91)	5.27 (1.02)	5.21 (1.01)	1.65 (0.18)
Triglyceride (mmol/L): M (SD)	1.29 (0.82)	1.35 (0.96)	1.50 (1.16)	1.77 (1.15)	17.99 (< **0.001**)	1.47 (1.38)	1.59 (0.80)	1.90 (1.26)	2.15 (1.16)	33.14 (< **0.001**)
HDL (mmol/L): M (SD)	1.51 (0.47)	1.44 (0.38)	1.38 (0.38)	1.32 (0.35)	16.18 (< **0.001**)	1.64 (0.41)	1.54 (0.38)	1.48 (0.38)	1.34 (0.32)	52.97 (< **0.001**)
LDL (mmol/L): M (SD)	3.08 (0.87)	3.00 (0.79)	3.19 (0.82)	3.18 (0.86)	4.83 (**0.002**)	3.39 (0.85)	3.42 (0.85)	3.52 (0.94)	3.52 (0.90)	2.78 (**0.04**)
Glucose (mmol/L): M (SD)	6.44 (2.62)	5.65 (1.76)	6.44 (2.62)	5.65 (1.76)	15.24 (< **0.001**)	5.88 (2.17)	5.69 (1.75)	5.86 (1.78)	5.88 (1.55)	1.33 (0.26)
HbA1c (%): M (SD)	6.62 (1.62)	6.31 (1.15)	6.30 (1.06)	6.20 (0.83)	8.04 (< **0.001**)	6.42 (1.30)	6.30 (0.98)	6.38 (0.94)	6.52 (1.03)	3.08 (**0.03**)
Creatinine (umol/L): M (SD)	73.72 (12.58)	81.19 (12.95)	84.63 (14.97)	94.63 (24.90)	86.95 (< **0.001**)	62.04 (11.55)	66.52 (12.25)	70.35 (16.03)	83.30 (28.97)	135.94 (< **0.001**)
CRP (mg/L): M (SD)	2.20 (5.32)	1.90 (4.25)	1.85 (2.81)	2.55 (5.06)	2.97 (**0.03**)	1.97 (5.76)	2.15 (5.13)	2.58 (4.68)	2.92 (3.98)	3.49 (**0.02**)

*Bold values are statistically significant*.

### Incidence of Cardiometabolic Disease in Each Quartile

During the follow-up visits, it was found that 342 men (7.4%) and 360 women (8.6%) had developed hypertension; 98 men (2.48%) and 135 women (3.06%) had developed diabetes; and 175 men (5.04%) and 214 women (4.51%) had developed metabolic syndrome. Incident hypertension, and metabolic syndrome and diabetes increased with the increased UA levels at baseline (*P* < 0.001).

### Cox Regression Analysis for Cardiometabolic Disease

The Cox regression analysis using cardiometabolic disease as the dependent variable and the UA quartile as the independent variable was used to evaluate the association between serum UA and cardiometabolic disease (Table 3). Confounding factors, namely, SBP, TG, TC, HDL, LDL, Cr, CRP, glucose, and BMI were included in the analysis for men, and SBP, TG, HDL, LDL, Cr, CRP, and BMI were included in the analysis for women. The analysis showed that a high level of serum UA was associated with an increased incidence of diabetes, hypertension, and metabolic syndrome in men. Hazard ratios (HRs) (95% CI) were 2.08 (0.89–4.85) for M4 for diabetes, 1.72 (1.07–7.770) and 1.54 (0.94–2.51) for M3 and M4 for hypertension, and 1.61(0.95–2.72) and 1.61 (0.93–2.77) for M3 and M4 for metabolic syndrome. An increased level of UA was associated with an increased incidence of diabetes and metabolic syndrome in women; the HRs (95%) were 1.50 (0.95–2.36) and 1.56 (0.94–2.56) for F3 and F4 for diabetes, and 1.28 (0.96–1.70) for F4 for hypertension. Interestingly, the high levels of UA in M3 and M4 were associated with a reduced HR for metabolic syndrome in women.

**Table 3:** Risk of disease by categories of serum uric acid of baseline (raw percentages) (uric acid is from baseline. All diseases are from 2017 follow-up data).

**Variables**	**Serum uric acid quartile(mg/dL) (Men)**				**Serum uric acid quartile(mg/dL) (Women)**			
	**≤4.52**	**4.53–5.34**	**5.35–6.33**	**≥6.34**	**≤4.52**	**4.53–5.34**	**5.35–6.33**	**≥6.34**
**Heart failure**								
No. of subjects: n (heart failure subjects)	97 (24)	152 (48)	205 (48)	256 (60)	331 (78)	249 (79)	212 (67)	133 (43)
No. of incidents of HF(rate: %)	42 (10.32)	80 (11.94)	106 (11.88)	124 (10.72)	141 (9.32)	122 (10.77)	107 (11.33)	89 (14.26)
Person-years	407	670	892	1,157	1,513	1,133	944	624
Crude 95% CI	1	1.37 (0.71–2.65)	0.87 (0.44–1.71)	1.21 (0.65–2.25)	1	1.15 (0.79–1.69)	1.09 (0.73–1.63)	1.14 (0.71–1.83)
Adjusted 95% CI	1	1.18 (0.60–2.32)	0.73 (0.36–1.47)	0.86 (0.43–1.72)	1	1.27 (0.86–1.88)	1.30 (0.85–1.99)	1.55 (0.90–2.67)
**Heart disease events**								
No. of subjects	104 (59)	164 (93)	227 (139)	279 (177)	353 (201)	265 (168)	225 (152)	141 (99)
No. of incidents of HD event (rate: %)	20 (4.23)	34 (4.55)	35 (3.28)	53 (4.14)	83 (5.13)	55 (4.45)	63 (6.26)	28 (4.05)
Person-years	473	748	1,067	1,280	1,618	1,236	1,006	692
Crude 95% CI	1	0.77 (0.34–1.71)	0.90 (0.44–1.86)	0.77 (0.37–1.57)	1	0.88 (0.55–1.39)	0.70 (0.42–1.17)	0.61 (0.32–1.18)
Adjusted 95% CI	1	0.80 (0.35–1.82)	0.93 (0.43–1.99)	0.79 (0.35–1.77)	1	0.91 (0.57–1.46)	0.77 (0.44–1.33)	0.73 (0.35–1.53)
**Atrial fibrillation**								
No. of subjects	101 (3)	159 (5)	221 (16)	272 (13)	343 (18)	262 (9)	222 (6)	137 (8)
No. of incidents of AF (rate: %)	5 (1.05)	7 (0.92)	19 (1.81)	24 (1.84)	28 (1.7)	16 (1.26)	16 (1.51)	17 (2.47)
Person-years	474	765	1,051	1,302	1,648	1,270	1,061	687
Crude 95% CI	1	0.66 (0.13–3.26)	1.52 (0.42–5.51)	1.08 (0.29–4.01)	1	0.76 (0.30–1.93)	0.73 (0.28–1.95)	1.71 (0.70–4.19)
Adjusted 95% CI	1	0.66 (0.13–3.35)	1.74 (0.45–6.75)	1.49 (0.35–6.23)	1	0.80 (0.31–2.09)	0.80 (0.28–2.28)	2.19 (0.78–6.19)
**Diabetes**								
No. of subjects	101 (26)	162 (39)	216 (62)	273 (74)	346 (91)	259 (78)	224 (69)	139 (46)
No. of incidents of DM (rate: ‰)	7 (1.49)	15 (1.93)	32 (3.10)	44 (3.41)	38 (2.29)	36 (2.90)	36 (3.44)	25 (3.61)
Person-years	470	776	1,033	1,290	1,656	1,242	1,047	692
Crude 95% CI	1	1.30 (0.53–3.18)	2.07 (0.91–4.69)^*^	2.28 (1.03–5.06)^*^	1	1.26 (0.80–1.99)	1.50 (0.95–2.36)^*^	1.56 (0.94–2.59)^*^
Adjusted 95% CI	1	1.29 (0.52–3.21)	1.98 (0.85–4.58)	2.08 (0.89–4.85)^*^	1	1.12 (0.70–1.78)	1.29 (0.80–2.08)	1.24 (0.70–2.21)
**Hypertension**								
No. of subjects	97 (35)	154 (68)	216 (102)	274 (137)	337 (150)	259 (124)	218 (103)	130 (72)
No. of incidents of Hypertension (rate: ‰)	23 (5.19)	44 (6.15)	86 (8.90)	96 (7.75)	121 (7.75)	86 (7.34)	94 (9.75)	59 (9.38)
Person-years	443	715	966	1,239	1,562	1,172	964	629
Crude 95% CI	1	1.17 (0.71–1.94)	1.68 (1.06–2.66)^*^	1.47 (0.94–2.32)^*^	1	0.94 (0.71–1.24)	1.24 (0.95–1.63)	1.17 (0.85–1.59)
Adjusted 95% CI	1	1.20 (0.72–1.99)	1.72 (1.07–2.77)^*^	1.54 (0.94–2.51)^*^	1	1.00 (0.76–1.33)	1.28 (0.96–1.70)^*^	1.18 (0.82–1.68)
**Metabolic syndrome**								
No. of subjects	95 (29)	148 (56)	202 (83)	256 (125)	323 (196)	239 (165)	207 (169)	123 (102)
No. of incidents of Mt Syndrome (rate: %)	20 (4.48)	29 (4.03)	58 (6.04)	68 (5.60)	99 (6.40)	57 (4.95)	42 (4.21)	16 (2.49)
Person-years	446	719	960	1,214	1,546	1,152	998	643
Crude 95% CI	1	0.90 (0.51–1.59)	1.35 (0.81–2.25)	1.26 (0.76–2.07)	1	0.77 (0.56–1.07)	0.66 (0.46–0.95)^**^	0.39 (0.23–0.66)^**^
Adjusted 95% CI	1	1.01 (0.57–1.80)	1.61 (0.95–2.72)^*^	1.61 (0.93–2.77)^*^	1	0.96 (0.69–1.35)	0.84 (0.58–1.23)	0.56 (0.31–1.01)^*^
**Kidney disease(eGFR)**								
No. of subjects	98 (13)	160 (38)	215 (65)	269 (119)	346 (81)	256 (93)	221 (93)	134 (70)
No. of incidents of kidney disease (rate: %)	20 (4.52)	35 (4.78)	37 (3.65)	71 (5.77)	70 (4.39)	65 (5.49)	53 (5.28)	33 (4.99)
Person-years	442	732	1,014	1,230	1,596	1,185	1,004	661
Crude 95% CI	1	1.06 (0.61–1.83)	0.80 (0.46–1.38)	1.26 (0.77–2.08)	1	1.24 (0.88–1.74)	1.20 (0.84–1.71)	1.10 (0.73–1.66)
Adjusted 95% CI	1	1.03 (0.59–1.79)	0.78 (0.45–1.36)	1.23 (0.73–2.06)	1	1.18 (0.83–1.67)	1.03 (0.71–1.50)	0.84 (0.53–1.36)

* p < 0.10;

** *p < 0.05. Bold values are statistically significant*.

## Discussion

The results of the present study demonstrate that the incidence of heart disease after 4 years of follow-up was statistically significantly higher with increasing baseline levels of serum UA in community-dwelling older people. The fact that this incidence is significant even after adjusting for confounding factors suggests that UA is an independent predictor of the risk of cardiometabolic disease in this population.

Our cohort comprises people aged 60 years and above, and the average age of both women and men was 70 (6) years. Our results suggest that serum UA is a significant risk factor for cardiometabolic disease in older adults, independent of other risk factors. The association between UA and cardiometabolic disease has been examined in previous cohort studies, which have shown consistent findings demonstrating that increased UA levels are associated with a significantly higher risk of cardiovascular mortality (16) and predicted cardiometabolic disease in middle-aged men without clinical cardiometabolic disease and diabetes (17). Our study extends these previous findings to confirm that increased UA levels are associated with a higher risk of cardiometabolic disease in community-dwelling older people without clinical cardiometabolic disease and diabetes. This is the first study to examine the association between UA and risk of cardiometabolic disease in a Chinese community-based older adult cohort.

The results on the association between elevated levels of serum UA and elevated levels of lipids, insulin, and CRP in our study are consistent with those of previous studies. The mechanism by which hyperuricemia is associated with risk factors for cardiometabolic disease may be due to several pathological and endocrine factors. First, elevated serum UA may be associated with endothelial and inflammatory function as a result of increased oxidative stress (18). Hyperuricemia may increase the production of oxygen free radicals and platelet adhesiveness, which may explain the association between hyperuricemia and coronary heart disease events (19). Hyperuricemia has been associated with reduced insulin sensitivity, as demonstrated in Facchini's study (20). It is possible that serum UA levels are associated with insulin resistance, and that the interaction with insulin resistance is at the level of the kidneys (20). Further, it has been demonstrated that high UA levels are independently associated with increased sodium resorption in men (21). This association may be a reason for hypertension. UA is strongly associated with increased incidence of metabolic syndrome, a key risk factor of cardiometabolic disease (22).

Our results indicate that when the serum UA level exceeded 6.34 mg/dl in males, new-onset diabetes increased significantly. When the serum UA level exceeded 5.35 mg/dl in males, the onset of hypertension in males increased significantly. When serum UA exceeded 5.35 mg/dl in males and 6.34 mg/dl in females, the onset of metabolic syndrome increased significantly. Similar results were also reported in previous studies on the relationship between UA levels and onset of cardiometabolic disease (23, 24). However, in these studies the UA level became a significant risk at much higher levels than 6.2 mg/dl in men (23). Possibly, the age of the participants in our study is much greater than the age of those in Takase's (23) and Verdecchia's studies (24) and even a small elevation to 5.34 mg/dl can lead to a higher risk of cardiometabolic disease in men. The second possible reason is that the men in our study were on medications for HTN, hyperlipidemia, or diabetes at baseline. That is, at baseline they were not healthy. These reasons may account for the range of variations in MetS components. Third, the case numbers in the high level of UA group in women reduced dramatically compared with the lower end of UA might contribute to these inconsistent results between men and women. Serum UA levels exceeding 5.34 mg/dl make UA a sensitive marker of kidney dysfunction and may cause a deterioration in kidney function (25). This decreased kidney function and low eGFR are associated with an increase in BP and the development of hypertension (9). Another potential mechanism may be the deposition of UA on the vascular wall as monosodium urate crystals, which would affect coagulation, and likely lead to arteriosclerosis and hypertension.

### Strengths and Limitations

This study is the first to investigate the relationship between serum UA levels and the development of cardiometabolic disease in older community-dwelling adults in China. However, it has several limitations. First, the study participants were invited to participate while undergoing health checks and hence may include those who are particularly concerned about their health, especially those who completed follow-up data collection. Second, the laboratory measures, including serum UA, were obtained using a single blood sample and continuous data collection was not conducted. Thus, the confounding effects of other factors subject to change, such as smoking, drinking, and diet, were not considered, and hence, the results of the study need to be interpreted with caution. Finally, serum cystatin C rather than serum creatinine should be measured in a future study to provide an accurate assessment of damage to the kidneys.

## Conclusion

UA is an independent predictor of new-onset diabetes and hypertension in both women and men, and a predictor of new-onset metabolic syndrome only in men.

## Data Availability Statement

All datasets generated for this study are included in the article/supplementary material.

## Ethics Statement

The studies involving human participants were reviewed and approved by the Ethics Committee of Shanghai East Hospital. The patients/participants provided their written informed consent to participate in this study.

## Author Contributions

HF, JL, and ZL contributed to the study conception, study design, interpretation of the data, and critical revision of the manuscript. QL contributed to the study conception, design, and data analysis. JS contributed to the data analysis, interpretation of the data, and drafting the manuscript. NB reviewed the manuscript. LZ, HW, XZ, FL, QM, XX, and AY contributed to the collection of the data. All authors read and approved the final manuscript.

### Conflict of Interest

The authors declare that the research was conducted in the absence of any commercial or financial relationships that could be construed as a potential conflict of interest.
